# Characterization of *Withania somnifera* Leaf Transcriptome and Expression Analysis of Pathogenesis – Related Genes during Salicylic Acid Signaling

**DOI:** 10.1371/journal.pone.0094803

**Published:** 2014-04-16

**Authors:** Modhumita Ghosh Dasgupta, Blessan Santhosh George, Anil Bhatia, Om Prakash Sidhu

**Affiliations:** 1 Division of Plant Biotechnology, Institute of Forest Genetics and Tree Breeding, R.S. Puram, Coimbatore, Tamil Nadu, India; 2 CSIR-National Botanical Research Institute, Rana Pratap Marg, Lucknow, Uttar Pradesh, India; Macquarie University, Australia

## Abstract

*Withania somnifera* (L.) Dunal is a valued medicinal plant with pharmaceutical applications. The present study was undertaken to analyze the salicylic acid induced leaf transcriptome of *W. somnifera*. A total of 45.6 million reads were generated and the *de novo* assembly yielded 73,523 transcript contig with average transcript contig length of 1620 bp. A total of 71,062 transcripts were annotated and 53,424 of them were assigned GO terms. Mapping of transcript contigs to biological pathways revealed presence of 182 pathways. Seventeen genes representing 12 pathogenesis-related (PR) families were mined from the transcriptome data and their pattern of expression post 17 and 36 hours of salicylic acid treatment was documented. The analysis revealed significant up-regulation of all families of PR genes by 36 hours post treatment except *WsPR10*. The relative fold expression of transcripts ranged from 1 fold to 6,532 fold. The two families of peroxidases including the lignin-forming anionic peroxidase (*WsL-PRX*) and suberization-associated anionic peroxidase (*WsS-PRX*) recorded maximum expression of 377 fold and 6532 fold respectively, while the expression of *WsPR10* was down-regulated by 14 fold. Additionally, the most stable reference gene for normalization of qRT-PCR data was also identified. The effect of SA on the accumulation of major secondary metabolites of *W. somnifera* including withanoside V, withaferin A and withanolide A was also analyzed and an increase in content of all the three metabolites were detected. This is the first report on expression patterns of PR genes during salicylic acid signaling in *W. somnifera*.

## Introduction

*Withania somnifera* (L.) Dunal belonging to family Solanceae is commonly known as Ashwagandha or Indian ginseng and is a valued medicinal plant with pharmaceutical and nutraceutical applications. It is widely used in traditional medical systems of India and Africa as an adaptogens or vitalizers. The phytochemical analysis of root and leaf tissues of this species has been extensively studied [1], [2] and the major metabolites reported include alkaloids (isopelletierine, anaferine), steroidal lactones (withanolides, withaferins), saponins (sitoindoside VII and VIII) and withanolides. These chemical components possess anti-inflammatory, anti-stress, antitumor, antioxidant, anti-aging, immunomodulatory properties, hemopoetic effect, rejuvenating effect and provide cardiovascular protection [3], [4], [5], [6]. In a recent report, the leaf and root transcriptome of *W. somnifera* was analyzed to elucidate the withanolide biosynthetic pathway [7].

Molecular signaling during plant defense response is widely documented and involves three major pathways including salicylic acid (SA) dependent pathway predominant during interactions with biotrophic pathogens and jasmonic acid (JA) and ethylene (ET) dependent pathways effective during necrotrophy and herbivory. Extensive cross talk between the pathways has been reported [8], [9], [10]. Other phytohormones like abscisic acid, gibberellins, auxins, cytokinins, and brassinosteroids are also documented to regulate plant immune response [11], [12] indicating that plant growth and defense are tightly linked [13].

SA is the key hormone during biotic defense response and levels of SA and its glycosylated conjugate (SAG) in tissues are known to drastically accumulate both locally and systemically after pathogen infection [14]. Additionally, blockade of SA, impairs deployment of systemic acquired resistance (SAR) [15]. The best characterized SA induced genes (SAIGs) include the pathogenesis – related (PR) gene families coding for proteins with antimicrobial activity [16], [17]. Studies at molecular level have indicated that the SAIGs are activated by transcriptional control rather than by increase in the mRNA levels [18]. An extensive reprogramming from primary to secondary pathways with down-regulation of non-essential cellular activities is also reported during SA signaling [19].

Exogenously application of SA was reported to enhance disease resistance and induce PR gene expression in a wide variety of plant species like sunflower, wheat, *Musa* sp. and pepper [20], [21], [22], [23]. Further, the expression of PR genes during host-pathogen interaction has been extensively documented in solanaceous species like tomato [24], [25], tobacco [26], potato [27], [28], [29] and *Capsicum* [30]. The accumulation of PR proteins/up-regulation of PR genes during host – pathogen interaction in woody perennials was reviewed and the predominantly reported PRs in trees included PR-1, PR-2, PR-3, PR-5, PR-9, PR-10 and PR-12 [31].

Transcriptome analysis to comprehend gene expression during pathogen infection was recently reported from several species including *Musa* sp. [32], [33], wheat [34], potato [35], *Arabidopsis* [36], peach [37], *Lactuca sativa* [38] and *Citrus* sp. [39], [40]. However, to our knowledge the transcriptome induced by SA in plants has not been reported. In the present study, the leaf transcriptome of *W. somnifera* during exogenous application of SA was characterized. The RNA-Seq approach employed in the present study to analyze the global expression of transcripts during SA signaling is the first report on understanding the SAIGs in this species.

## Materials and Methods

### Plant material

*Withania somnifera* seeds were germinated *in vitro* and axillary shoots from one month old plants were used as explants for micropropagation. Multiple shoot induction was done in MS media supplemented with 0.5 mg/L BA and cultures were incubated in 25±2°C, 40–50% relative humidity with photoperiod of 16 h light and 8 h dark conditions. The proliferated multiple shoots were maintained by regular sub-culturing every 4–5 weeks.

### Exogenous application of salicylic acid (SA)

Initially, the optimization of SA concentration for exogenous application was conducted by incubating the leaf discs from *in vitro* raised plantlets to different concentration of SA (5 mM, 10 mM and 20 mM) at room temperature. A control of sterile water treatment was included to document the elicitation of SA. Observations were made for appearance of yellowing and necrotic symptoms in both SA and water treated (control) leaf discs.

In subsequent experiments, 5 mM SA was spayed uniformly on the plantlets aseptically at every 12 hours for a total time period of 36 hours. The control treatment included exogenous application of sterile water on the plantlets. Two hours after the final treatment, plantlets were washed thoroughly with sterile water and leaves were excised and immersed in RNA stabilization reagent, RNA*later* (Qiagen, Hilden, Germany) for RNA isolation and transcriptome analysis.

The experiment on reference gene selection was conducted on leaves harvested from water treated (control) and SA spayed (treated) plantlets harvested 17 hours post application. Expression profiling of PR genes and estimation of secondary metabolites were conducted on SA sprayed leaves harvested from 17 and 36 hour post SA treatment against water treated control leaves harvested at 36 hour.

### RNA extraction, library construction and sequencing

Total RNA from 36 hour post SA treated leaves was isolated using Plant tissue total RNA extraction spin kit (Chromous Biotech Pvt Ltd, India) and RNA integrity was confirmed using the 2100 Bioanalyzer (Agilent Technologies Inc., Santa Clara, CA). Subsequently, TruSeq RNA Sample Preparation Kit (Illumina Inc., San Diego, CA, USA) was used for purification and fragmentation of RNA, cDNA synthesis, end repair and adapter ligation followed by enrichment with PCR to create a cDNA library suitable for cluster generation following manufacturer's protocol. The QC of the amplified library was determined using high sensitivity bioanalyzer chip (Agilent Technologies Inc., Santa Clara, CA). The sequencing of the cDNA library was performed on Illumina Genome Analyzer IIx sequencer and 72 base paired end sequencing was conducted.

### De novo assembly and sequence annotation

The raw reads generated were filtered for weak and low signals (mean quality score > = 20) followed by adaptor trimming using Trimmomatic read trimming tool for Illumina NGS data [41]. The high quality (HQ) reads were then assembled *de novo* into contigs with De-brujin graph based assembler Velvet 1.2.07 (http://www.ebi.ac.uk/~zerbino/velvet/) [42] on different kmers. The contig assembly was followed by transcriptome assembly with default parameters using Oases transcriptome Assembly pipeline 0.2.08 (http://www.ebi.ac.uk/zerbino/oases/) [43]. The *de novo* assembly validation was conducted using CLC Genomics Workbench (CLC Bio, Aarhus, Denmark). The functional annotation was performed by aligning the transcript contigs to non-redundant (Nr) database of NCBI using BLASTx for green plants (http://www.ncbi.nlm.nih.gov) with cut off E value 1e -^06^ to identify transcripts with significant similarity.

### Analysis and validation of transcript contig assembly

Gene ontology (GO) mapping of transcript contigs were performed to classify their functions and categorize them into biological, molecular and cellular functions [44]. The Accession IDs derived from BLASTx were directly searched in GO database. GO terms for annotated transcript contigs were retrieved using different databases including UniProtKB (http://www.uniprot.org/help/uniprotkb), TAIR (www.arabidopsis.org/) and Sol Genomics Network (SGN) (http://solgenomics.net/). The E-value distribution and sequence similarity distribution was determined to evaluate the success of the alignment for a given sequence database and the overall performance of the alignments, respectively. The experimental evidence for existence of the protein was determined through the Evidence Code (EC) distribution of the annotated transcript contigs. The annotation distribution graph was prepared to determine the number of GO terms assigned per sequence.

### Functional characterization and pathway analysis

The ortholog assignment and mapping of the transcript contigs to biological pathways were performed according to the Kyoto Encyclopedia of Genes and Genomes (KEGG) automatic annotation server (KAAS) [45]. All transcript contigs were compared against the KEGG database using BLASTx, with default threshold bit –score value of 60.

### Identification of simple sequence repeats (SSRs)

All transcript contigs in the draft assembly were analyzed for presence of SSRs using MISA standalone SSR tool (http://pgrc.ipk-gatersleben.de/misa). SSR motifs from di- to hexa-nucleotide were identified with the criteria of atleast 6 repeats for di- and five repeats for tri-, tetra-, penta- and hexa- nucleotide.

### Discovery of miRNAs

The potential conserved miRNAs in the transcriptome data was identified by mapping the transcript contigs against known plant hairpin (5,077) and mature (5,855) miRNA sequences deposited in miRBase version 19 (http://www.mirbase.org/) using CLC Genomic Work bench [46].

### Selection of reference genes for normalization of qRT-PCR data

Expression profiling of transcripts in biological systems using qRT-PCR obligates the use of a stable reference or house-keeping gene for normalization of data. In the present study, a set of six commonly used reference genes, viz., 60 S ribosomal protein L2 (*WsRPL*), actin (*WsAct*), glyceraldehyde-3-phosphate dehydrogenase (*WsGAPDH*), α-tubulin (*WsTUB*), ADP-ribosylation factor (*WsARF*) and histone H2B (*WsH2B*) were mined from the transcriptome data and analyzed for its suitability as a reference gene for the given tissue and experimental condition. Primer pairs were designed using Beacon Designer (http://www.premierbiosoft.com/molecular_beacons), analyzed using BLASTn and Primer-BLAST (www.ncbi.nlm.nih.gov/tools/primer-blast) to ensure specificity and were subsequently used in qRT-PCR analysis (table 1).

**Table 1:** Details of primer pairs and amplicon size of reference genes used for normalization of qRT-PCR data in *W. somnifera.*

Gene Name and ID	Sequence of Primer pairs	Amplicon length (bp)	Tm (°C)
	Forward Primer Sequence 5′-3′		
	Reverse Primer Sequence 5′-3′		
60 S ribosomal protein L2 (*WsRPL*)	GAGGACGTACTGAGAAACCTATG TACTAGCATGACCAATGTGTTGA	156	79.4
Actin (*WsAct*)	AGATATTCAGCCTCTTGTCTGTG ATTGAGCCTCATCACCAACATA	170	81.1
Glyceraldehyde-3-phosphate dehydrogenase (*WsGAPDH*)	ATGCTCAAGTATGATTCCACTCA GAAGACACCAGAAGATTCAACAAC	174	78.2
α-Tubulin (*WsTUB*)	AAATGCTTGCTGGGAACTTTAC TCCTGTCCTCACTTCATCAATG	193	82.2
ADP-ribosylation factor (*WsARF*)	GAGATTGTTACCACTATTCCTACCA CACGATCACGATCATTACTATCAAC	178	78.6
Histone H2B (*WsH2B*)	TTCTAGCAAGTCAATGGGTATCAT CTTTAGTTCCTTCAGAAACAGCAT	188	78.1

Leaves were harvested from water treated (control) and SA treated plantlets (as described earlier) after 17 hours post treatment and stored at −80°C until further use. Total RNA was isolated individually from all tissues using Plant tissue total RNA extraction spin kit (Chromous Biotech Pvt Ltd, India) using manufacturer's protocol. The qRT-PCR reactions were performed in fast optical reaction tube (Microamp- Applied Biosystems, USA) and a 20 µl reaction included 50 ng of cDNA, 10.0 µl SYBR Green JumpStart Taq ReadyMix (Sigma, St. Louis, MO, USA), 150 nM each of forward and reverse primer and 0.8 µl of 20 mg/ml BSA. All reactions were conducted as three independent technical replicates in ABI PRISM 7500 Step One plus Sequence Detection System (Applied Biosystems, USA) using the following program - one cycle of 95°C for 10 min; 40 cycles of 95°C for 15 sec and 60°C for 1 min. The melting curve was determined for each primer pair to confirm the specificity of the amplified product.

### Statistical analysis for stability of gene expression

The expression level and stability of the six selected endogenous genes were evaluated with statistical programs including geNorm [47] and Normfinder [48] downloaded from GenEX standard software (http://GenEx.gene-quantification.info) and BestKeeper, an Excel-based tool [49] downloaded from (http://www.gene-quantification.de/bestkeeper.html). Expression levels were assessed based on the number of amplification cycles needed to reach a fixed threshold (Cycle threshold - Ct) in the exponential phase of PCR.

Ct values were imported to GenEX software and analyzed using geNorm and Normfinder tools following the developer's instruction. In geNorm analysis, the data in logarithmic value was automatically converted to linear scale and the gene pair with lowest M value (average expression stability value) was considered to have most stable expression [47]. NormFinder uses data in the logarithmic scale and takes into consideration information of groupings of samples and predicts the optimal reference based on the variability values [48]. In BestKeeper, the average Ct values were used to analyze the stability value of the candidate genes. BestKeeper creates a pair-wise correlation coefficient between each gene and generates the BestKeeper index as well as calculates coefficient of variance (CV) and the standard deviation (SD) of the Ct values using the whole data set [49]. Genes that exhibited the lowest coefficient of variance and standard deviation (CV±SD) were identified as the most stable gene. Genes that show a SD greater than 1 were considered unstable [50].

### Data mining for PR genes and expression profiling during SA signaling

The annotated transcriptome data was manually mined for pathogenesis –related genes [16] and the identity was confirmed by BLASTx analysis (table 2). The transcript contigs from each PR gene families were individually aligned using Clustal W2 (http://www.ebi.ac.uk/Tools/msa/clustalw2/) and distinct genes having pair-wise score less than 70 from each family were selected for expression profiling. Primer pairs for 17 genes representing 12 PR families were designed and synthesized (IDT Technologies, Canada) for qRT-PCR (table 2).

**Table 2:** Details of primer pairs and amplicon size of pathogenesis - related genes used for expression profiling during SA signaling.

S. No.	PR Family	Name	Sequence similarity	Primer Pairs	Amplicon size (bp)	Tm (°C)
1	PR-1	Pathogenesis- related protein PR-1 (*WsPR1*)	*Capsicum annum* (AY560589.1)	TGATGAGAAGCAATGGTATGACTAT CGATCAGACATCAGTTGGAAGT	183	79.8
2	PR-2	β-1,3-glucanase (*WsB13G*)	*Solanum tuberosum* (JX838875.1)	ACATTGCTTCGTCTATCAAAGTTTC CACCATGAGGTAAGAACCAGTT	158	78.5
3	PR-3	Class I chitinase (*WsCHTN1)*	*Solanum tuberosum* (U02605.1)	CTCAATCACCAAAGCCATCTTG AATTCCGCAGTACCTTCTGTAAA	194	81.9
4		Class II chitinase (*WsCHTN2*)	*Solanum tuberosum* (U49969.1)	CACAAGACAACAAGCCATCATG TAGAATCCAATTCGATCATCCACTT	175	80
5		Class IV chitinase (*WsCHTN4*)	*Nicotiana tabacum* (AB267862.1)	CTTCAAGCAATAATGGAGGTTCAG CTCACGCTTAGAATCATCAGTAGA	185	76.1
6	PR-5	Thaumatin-like protein (*WsTHAU*)	*Solanum lycopersicum* (XM_004230458.1)	ACGTCTTTGACACCGATGAATA ACATAGTCAGTAGAAGAGCAAGTG	166	78.1
7	PR-6	Cystatin-like protein (*WsCYST*)	*Solanum tuberosum* (DQ191655.1)	GTTGAAGATGGTCCTACCTTTACT CCTCAGCATTAGCATGAACAATC	196	79.4
8		Serine protease inhibitor like protein (*WsSPI*)	*Solanum lycopersicum* (XP_004234308.1)	ATGCCCGTCAAATTCATTAAGTTT TCCTCCAGTCTCCAACAATCTA	171	76.5
9	PR-8	Class III chitinase (*WsCHTN3*)	*Capsicum annum* (AB267862.1)	GAACTTGGATCACCACTTCATTAC GTAGTGAACTGACATGGAGGATT	197	77.1
10	PR-9	Lignin-forming anionic peroxidase (*WsL-PRX*)	*Solanum lycopersicum* (XM_004250354.1)	TCCACATTCTATGATCGCACTT AACGCAGTCTTCTCACTAACAA	194	78.3
11		Suberization-associated anionic peroxidase (*WsS-PRX*)	*Solanum tuberosum* (AAA33837.1)	GTGCAAAGAGAAATTCAGACAAGT AGAATACCTCCATCACAACCATC	175	80.5
12	PR-10	PR-10 type pathogenesis-related protein (*WsPR10*)	*Nicotiana tabacum* (AB518291.1)	AGTTGCTCATATAGAAGTCAAGTGT TCCATCATAGTTCAATCTCCATTCA	169	76.2
13	PR-11	Chitinase, class V (*WsCHTN5*)	*Nicotiana tabacum* (X77110.1)	TGCGAACAATCATGGTCTTAGA TCCTGAGTAACAATAATCTCCAACA	163	78.4
14	PR-12	Defensin (*WsDFSN*)	*Capsicum annum* (X95730.1)	TGCTGGTTTTTGCTACTGAGGCA CAGAAGCAACGGCGACGGAATC	151	81.4
15	PR-14	Nonspecific lipid transfer protein 2 (*WsLTPa)*	*Capsicum annum* (AY496100.1)	GTTGTGGTGGAGTTAAGAATTTGAT GGGCTGATCTTGTAAGGAATGT	169	80.3
16		Non-specific lipid-transfer protein-like protein At2g13820-like (*WsLTPb*)	*Solanum lycopersicum* (XP_004229337.1)	GCACTTCAACTCAATGTTACACTT CAACAGAAGGAATGGGACTATTTG	193	79
17	PR-16	Germin-like protein subfamily 1 member 20 (*WsGER1-20*)	*Solanum tuberosum* (AFW90592.1)	TAATGGCTGTGGTGACTTCAATA GGCATATTCAAACCCGATTTAAAGA	169	78.4

Total RNA was isolated from leaves harvested from 17 and 36 hours SA treated plantlets while leaves harvested from 36 hour post water treated plantlets were used as control. qRT-PCR reactions were conducted as described earlier for reference gene selection. The melting curve was determined for each primer pair to confirm the specificity of the amplified product and all reactions were conducted in three independent technical replicates. The qRT-PCR data was analyzed using the ΔΔCT method described by Livak and Schmittgen [51]. While calculating the ΔΔCT, undetermined Ct values were imputed to 40 [52] and fold decrease was calculated as the reciprocal of the fold change [53].

#### Statistical analysis of data

The fold expression of transcripts between control and SA treated cDNA pools were statistically analyzed by T-Test using SPSS software (version 20.0) and difference between treatments were considered statistically significant when P<0.01.

### Quantification of secondary metabolites during SA signaling

Leaves were harvested from water treated (control) and SA treated plantlets (as described earlier) after 17 and 36 hours post treatment and air dried. The samples were subsequently ground into fine powder and used for secondary metabolite analysis.

#### Extraction of metabolites

Three hundred mg of each leaf samples were extracted directly with chloroform hexane using a tissue homogenizer (Kinematica Polytron Homogenizer PT 6100). All solvents used for the extraction were of HPLC grade (Qualigen fine chemicals, India). The solvent portion was collected by filtration and the process was repeated until the chloroform layer was almost colourless. The combined extracts were filtered and the filtrate was concentrated under reduced pressure using rotovap (Laborota 4000, Heidolph, Germany) followed by high vacuum drying (EZ-2, Genevac, USA) to remove traces of solvent. Subsequently, the samples were lyophilized and stored at −20°C for analysis of metabolites. The dried samples were later dissolved (3 mg/ml) in methanol and filtered through 0.45 µm filter and degassed for one minute. The external standards used in HPLC analysis included withanosides-V, withaferin-A and withanolide-A (Natural Remedies Pvt. Ltd., Bangalore, India). The stock solutions of external standards were prepared in methanol at the concentration of 1 mg/mL.

#### HPLC analysis of secondary metabolites

The estimation of the three metabolites were performed on a Waters liquid chromatograph equipped with a Waters 600 controller, a Waters Delta 600 solvent delivery system, a Rheodyne 7125 sample injector fitted with a 20 µL loop, and a Waters 2996 Photodiode Array Detector, with Waters Empowered 2.154 software. A Supelco 516 C_18_ (4.6 mm×25 cm) reverse phase analytical column equipped with a Waters µBondapak C_18_ 10 µm precolumn was used for estimation. The wavelength scan range of the PDA was set to 190–350 nm and the presence of withanosides-V, withaferin-A and withanolide-A was detected at 227 nm. A. The isocratic mobile phase consisted of 60% acetonitrile containing 0.1% acetic acid (solvent A) and 40% water containing 0.1% acetic acid (solvent-B) at a flow rate of 1.0 mL min^−1^. The metabolites were estimated in comparison to the external standards and the results were presented as µg mg^−1^ of dry weight of leaf tissue.

## Results

### Optimization of SA treatment

Leaf discs subjected to different concentrations of SA showed varying levels of necrotic symptoms. Yellowing of leaf discs was observed in all treatments by one hour of SA application and complete necrosis was observed in 20 mM SA treatment. Hence, the optimal concentration was selected as 5 mM (Figure S1), which was used for further experiments.

**Figure 1:**
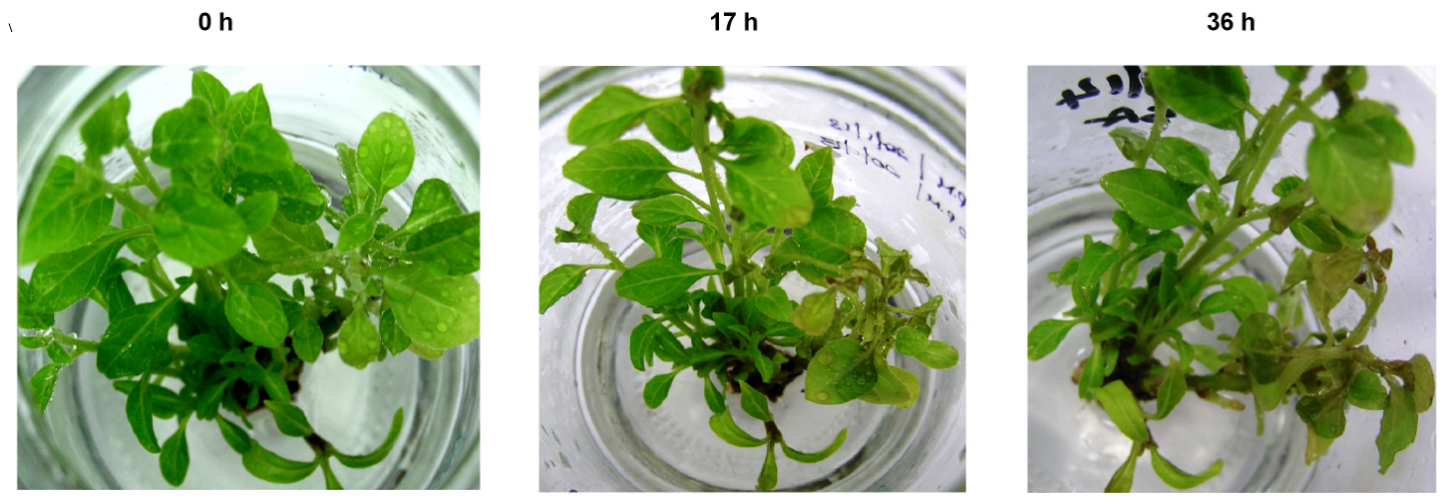
Effect of salicylic acid on *in vitro* grown plantlets of *W. somnifera*. 0 =  Plantlets immediately after application of SA; 17 h =  Plantlets after 17 hours post SA treatment; 36 h =  Plantlets after 36 hours post SA treatment.

Subsequently, plantlets were sprayed with 5 mM SA in intervals as described earlier and a control with plantlets sprayed with sterile water was maintained, to document the effect of SA. The SA treated plantlets showed initial symptoms of yellowing by 17^th^ hours and by 36^th^ hours the symptoms were prominent (Figure 1). No symptoms were observed in water treated control plantlets.

### Transcriptome sequencing and Read Statistics

Total RNA was isolated from leaves harvested from 36 hours post SA treated plantlets. The RNA was quantified and 10 µg of total RNA at a concentration of 400 ng/µl with OD260/280 = 1.8, RNA 28 S:18 S≥1.0 and RNA Integrity Number (RIN) of 7.0 was used for cDNA library construction. The cDNA library was sequenced using Illumina Genome Analyzer IIx Sequencer. The raw paired - end - sequence data was deposited in NCBIs Short Read Archive with the accession number SRA107547. A total of 45.6 million, 72 base paired – end reads (3.28 Gb) was generated. The raw reads were subjected to quality control and the total number of HQ reads was 87.26% (39.8 million reads).

### De novo assembly and functional annotation

**Figure 2:**
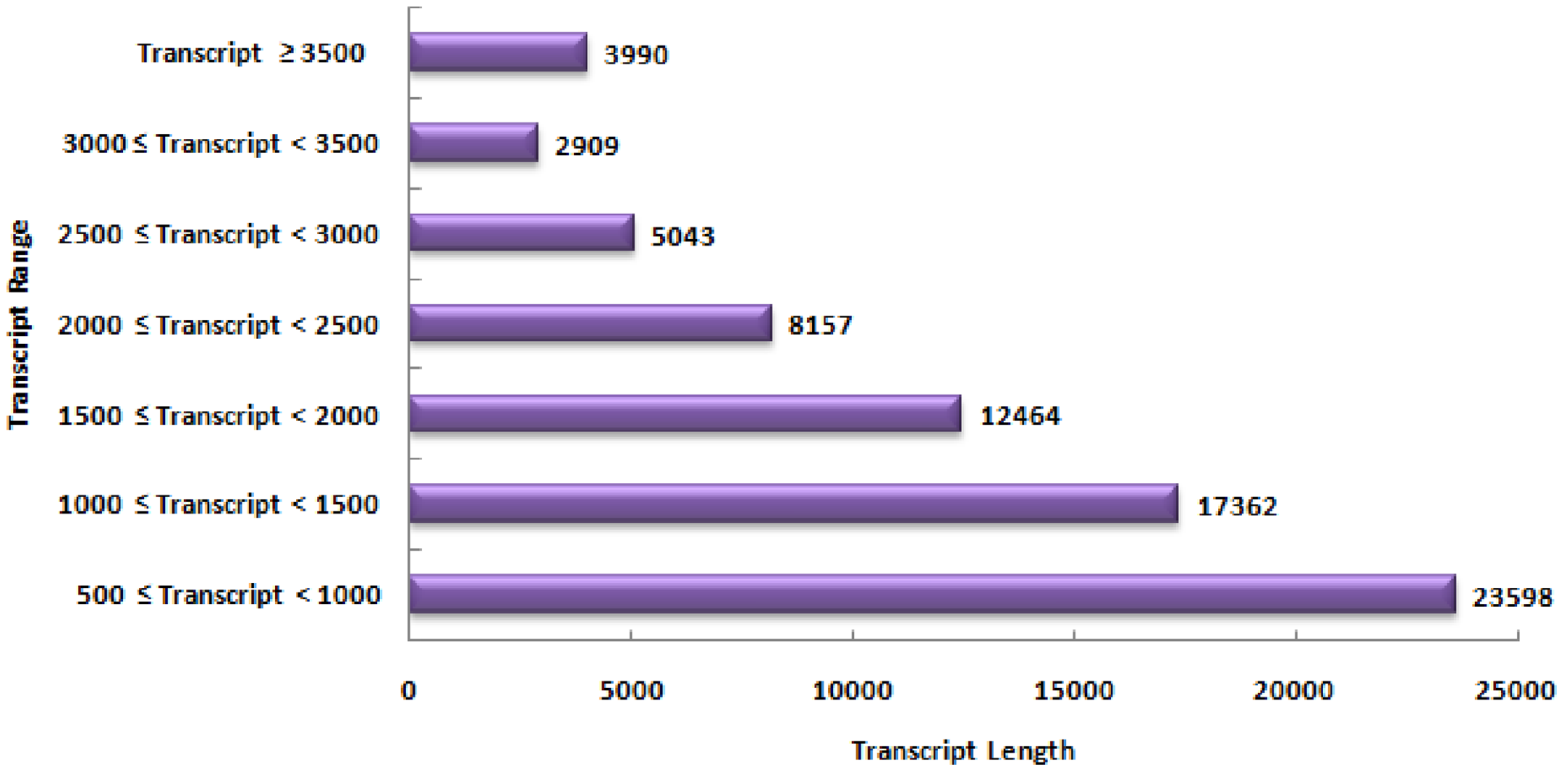
Distribution of transcript contig length in RNA-Seq data of *W. somnifera*.

The *de novo* assembly generated 73,523 transcript contigs with average transcript contig length of 1620 bp and the maximum length of contig transcript was 9489 bp. The total number of bases in transcript contigs was 119,136,311 bases (1.19 Gb). The distribution of transcript contig length is shown in figure 2. N50 (the smallest contig size in which half the assembly is represented) is the statistics used to assess the quality of sequence assembly and higher values suggest better assembly. In the present study the N50 was determined to be 1,978 bp. The assembled transcript contigs were annotated using BLASTx against Nr database for Viridiplantae (table S1). A total of 71,062 (96.65%) transcript were annotated while 2,461 transcripts had no significant BLAST hits. Maximum percent of *W. somnifera* sequences showed significant similarity with *Vitus vinifera*, followed by *Populus trichocarpa* and *Ricinus communis*. The E- value distribution and sequence similarity distribution are provided as Figure S2 and S3. The sequence similarity distribution revealed that about 80% of transcript contigs had positive alignment length which ranged from 50–90 percent.

### Gene Ontology (GO) Classification

The annotated transcript contigs were mapped on GO database and 53,424 (75%) sequences were assigned GO terms while 17,683 were not assigned any functional categories. The GO terms for the annotated transcript contigs were retrieved from different databases and maximum terms could be derived from UniProtKB followed by TAIR (Figure S4). The evidence code distribution for sequences and their translated products showed an over-representation of electronic annotations (IEA), although other non-automatic codes, such as inferred from direct assay (IDA), inferred from sequence or structural similarity (ISS) and inferred by mutant phenotype (IMP) were also well represented (Figure S5A and S5B). The annotation score distribution revealed that maximum number of transcript contigs annotated with 55–70 percent similarity (Figure S6).

**Figure 3:**
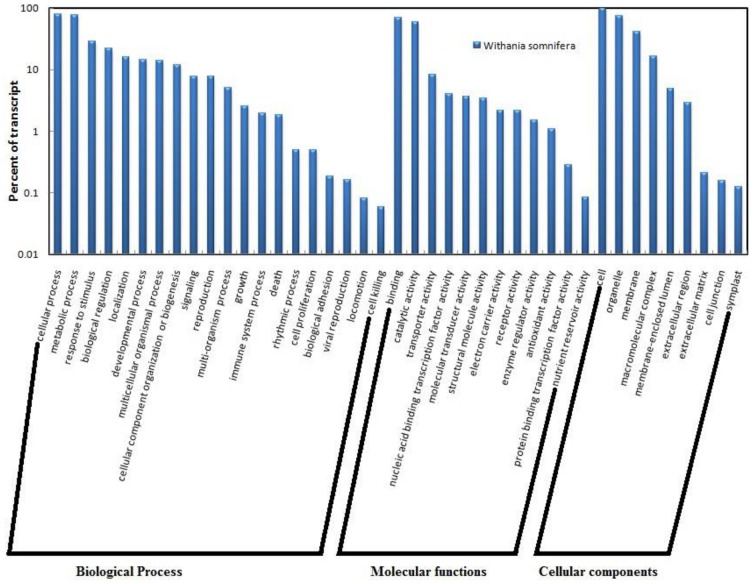
Gene Ontology classification of transcript contigs grouped under biological processes, molecular functions and cellular components.

The GO terms were grouped into different levels for all the three ontology domains i.e., biological processes, molecular functions and cellular components (figure S7). The number of transcript contigs categorized under biological function was 37,831 while 44,216 transcript contigs grouped under molecular functions. The cellular components clustered 38,312 transcript contigs. Each transcript contigs could be multi-functional and hence can lie in more than one GO term. The “metabolic process” and “cellular process” constituted the main biological processes in SA treated leaves of *W. somnifera* with 70% transcript contigs grouping to these ontologies. The main “metabolic function” included primary metabolic processes, biosynthetic processes, nitrogen metabolism while the “cellular process” included response to stimulus, cellular development process, cellular localization, cell communication and cell adhesion. “Catalytic activity” including transferase, hydrolase, oxidoreductase, ligase, lyase and isomerase activity were the main molecular functions in SA treated leaf tissues of this species. The ‘binding activity’ including protein, nucleotide, lipid and cofactor binding accounted for about 55% of the molecular functions. The cellular component represented by 38,312 transcript contigs mainly included genes involved in cell function (99%) followed by transcripts related to ‘organelle’ functioning (7%) (figure 3).

### Pathway annotation using KEGG

**Figure 4:**
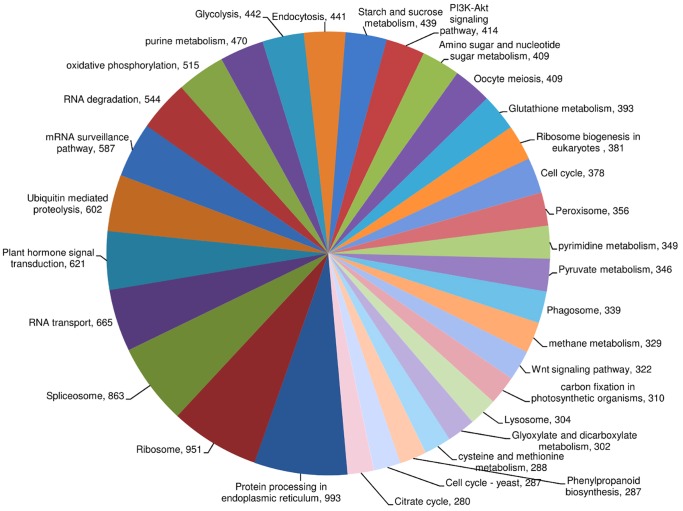
Distribution of transcript contigs to different biological pathways. Values indicate number of transcript contig representation in each pathway. Pathways with number of transcript contigs below 280 are not shown in the chart.

Ortholog assignment and mapping of transcript contigs to biological pathways were performed using KEGG (table S2). The annotated transcript contigs were assigned to 182 pathways and the major representation of transcript contigs was from protein processing in endoplasmic reticulum [PATH: ko04141; 993 transcript contigs] followed by ribosome [PATH: ko03010; 951 transcript contigs], spliceosome [PATH: ko03040; 863 transcript contigs], RNA transport [PATH: ko03013; 665 transcript contigs] and plant hormone signal transduction [PATH: ko04075; 621 transcript contigs] (Figure 4).

### Identification of SSRs

The leaf transcriptome data of *W. somnifera* generated a total of 4,250 SSRs with maximum representation of tri-nucleotide SSRs (2457) followed by di-nucleotide (1576), hexa-nulceotide (116) and tetra-nucleotide (86). Minimum number of SSRs (15) was registered under the category of penta-nucleotide.

### Identification of miRNAs

Hairpin and mature miRNAs in *W. somnifera* leaf transcriptome was identified by searching the public miRNA database. A total of 911 miRNAs were identified including 51 hairpin and 860 mature plant miRNAs. The mature miRNAs were distributed across 101 families and included isoforms found in various plant species. The largest family was miR169 with 18 members followed by miR171 (14 members), miR166 (12 members) and miR160 (9 members). Further, the family of miR393 and miR395 constituted eight members each.

### Selection of reference gene for normalization of qRT-PCR data

Gene expression stabilities of six genes including *WsRPL*, *WsAct*, *WsGAPDH*, *WsTUB*, *WsARF* and *WsH2B* were analyzed for their suitability in normalization of qRT-PCR data. The melt curve analysis of the six reference genes is provided in figure S8. The identification of the most stable reference gene was statistically derived using three independent programs. In geNorm analysis, *WsTUB* and *WsRPL* produced the lowest M value (0.21) while *WsH2B* had the highest M value (1.01) indicating that *WsTUB* and *WsRPL* had the most stable expression and *WsH2B* was the least stable. Normfinder analysis revealed *WsRPL* (0.11) as the best reference gene with lowest variability value followed by *WsTUB*. *WsH2B* (2.12) was predicted as the least stable. Similarly, in BestKeeper analysis *WsTUB* had a CV±SD value of 1.15±0.36, revealing highest stability followed by *WsRPL* (1.25±0.35). *WsARF* and *WsH2B* documented the least stability with values of 3.9±1.13 and 3.16±0.94 respectively. All the three programs revealed *WsTUB* as the most suitable reference genes for quantitative gene expression studies in *W. somnifera* during SA signaling. Hence, *WsTUB* was used for data normalization in subsequent experiments conducted on expression profiling of PR genes.

### Expression profiling of PR genes during SA signaling

**Figure 5:**
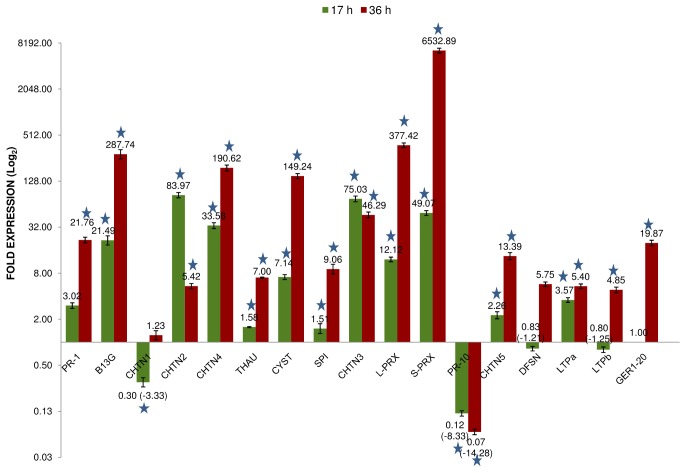
Expression profiling of Pathogenesis-related gene families in *W. somnifera*. Results are mean of triplicate data from independent replications and the error bars represent standard deviation. The gene IDs are given in Table-2 and the number on each bar represent relative fold expression. Asterisk indicate statistical significance of P<0.01. 17 h =  Expression of PR genes in 17 hours post SA treated leaf tissues; 36 h =  Expression of PR genes in 36 hours post SA treated leaf tissues.

The effect of SA on the temporal expression of 17 selected PR genes in leaves of *W. somnifera* was investigated across two time points (17 and 36 hours). The melt curve analysis of all the primer pairs revealed single product and absence of non-specific bands (figure S9). The expression of thirteen PR genes belonging to 10 families including PR1, chitinases (PR3, PR8, PR11), peroxidases (PR9), glucanase (PR2), thaumatin – like (PR5), cystatin (PR6), serine protease inhibitor (PR6), one member of lipid transfer protein (PR14) and germin-like (PR16) were up-regulated by 1.4 fold to 83 fold after 17 hours of SA treatment. The class II chitinase (*WsCHTN2*) documented maximum up-regulation by 83 fold followed by the class III chitinase (*WsCHTN3*) belonging to PR8 with 75 fold relative increase in expression when compared with its expression in water treated control leaf tissues. The expression of four genes including class I chitinase (*WsCHTN1*), *WsPR10*, defensin (*WsDFSN*) and one member of LTP (*WsLTPb*) were down-regulated post 17 hour SA treatment (figure 5). The down-regulation of *WsPR10* and *WsCHTN1* by 8 and 3 fold respectively was significant in comparison to other down-regulated transcripts.

The fold expression of all transcripts except *WsPR10* was up-regulated by 36 hours of SA treatment and the expression levels ranged from 1 fold to 6532 fold. Very high levels of expression was recorded for peroxidases with 377 and 6532 fold for *WsL-PRX* and *WsS-PRX* followed by glucanase with 287 fold, class IV chitinase (*WsCHTN4*) by 190 fold and cystatin (*WsCYST*) by 149 fold. The expression of class I chitinase (*WsCHTN1*), defensin (*WsDFSN*) and LTP (*WsLTPb*) which were slightly down-regulated after 17 hours of SA treatment, showed up-regulation by 1 to 5 fold after 36 hours of SA treatment. However, *WsPR10* continued to show down-regulation by 14 fold after 36 hours of treatment (Figure 5).

### Quantification of secondary metabolites during SA signaling

**Figure 6:**
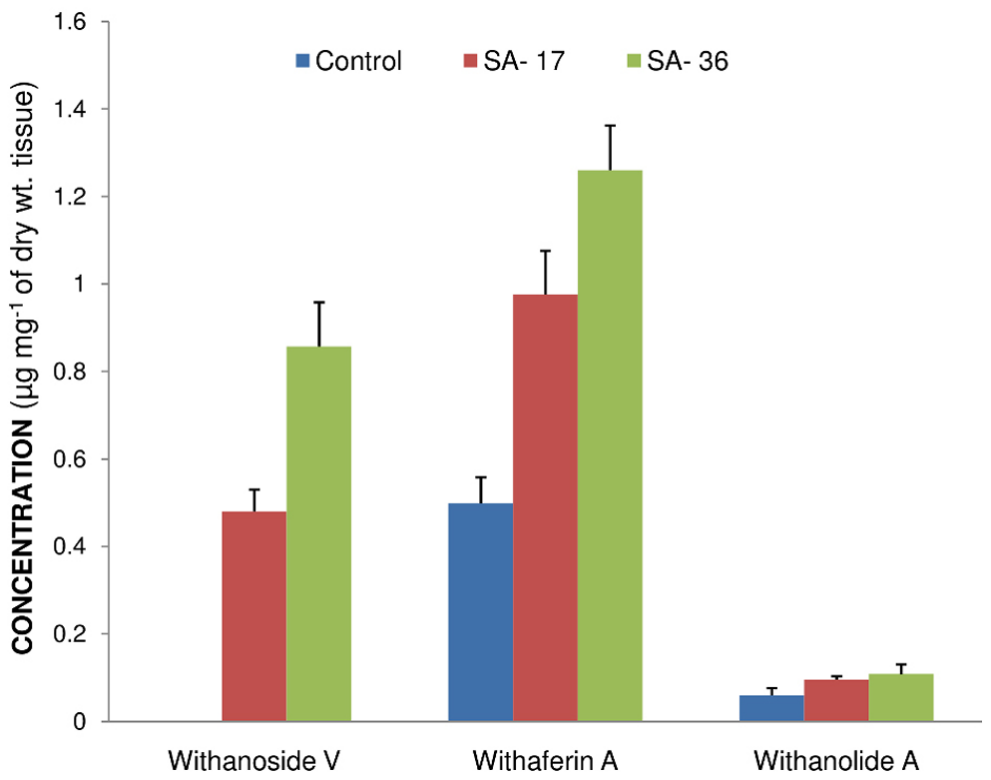
Quantitative variation in secondary metabolite content by exogenous application of salicylic acid. Control =  Metabolite content in water treated leaf tissues; SA-17 =  Metabolite content in 17 hours post SA treated leaf tissues; SA-36 =  Metabolite content in 36 hours post SA treated leaf tissues. Presence of withanoside V was not detected in water treated (control) leaf tissues.

The content of three major secondary metabolites including withanoside V, withaferin A and withanolide A in the leaf tissues of water treated control and 17 and 36 hour post SA treatment was estimated. An increase in the content of all the three metabolites was recorded. The maximum effect of SA was documented on withanoside V, with an increase in the content by 0.857 µg mg^−1^ in comparison to negligible levels in control. Withaferin A recorded an increase from 0.499 µg mg^−1^ in water treated control to 1.26 µg mg^−1^ in 36 hours SA treated leaf tissues. The withanolide A content was marginally increased by SA application (Table 3; figure 6).

**Table 3:** Estimation of secondary metabolites in control and salicylic acid treated leaf tissues of *W. somnifera*.

Standards	Leaf tissue samples		
	Control	SA-17	SA-36
**Withanoside V**	ND	0.4802±0.0500	0.8571±0.1010
**Withaferin A**	0.4985±0.0600	0.9755±0.1002	1.2598±0.1025
**Withanolide A**	0.0597±0.0164	0.0963±7.3000e-3	0.1087±0.0219

Mean values ±SD of µg mg^−1^ of dry weight of leaf sample; ND =  Not Detected; Control =  leaf samples from water treated plantlets; SA-17 =  leaf samples from 17 hours post SA treated plantlets; SA-36 = leaf samples from 36 hours post SA treated plantlets.

## Discussion

### Comparison of leaf transcriptome data in W. somnifera

The first report on *de novo* assembly, functional annotation and pathway analysis in *W. somnifera* leaf and root transcriptome was recently reported to identify putative genes involved in the withanolides biosynthesis [7]. The present study targeted the analysis of transcripts expressed in salicylic acid treated leaf tissues. The sequencing platforms used in both the studies were different wherein pyrosequencing was employed in the earlier study while Illumina platform was used in the present analysis. The number of transcript contigs annotated from the present assembly was 71,062, which was significantly higher than the transcripts annotated in the earlier study (38,961) using Nr database. The maximum number of transcripts annotated in the earlier report using four databases was 54,639 [7]. The probable reasons for over representation of transcripts in the present study could be attributed to the different assembly and annotation pipelines used across the two reports.

The functional classification and pathway assignment was performed using KEGG and a total of 124 pathways were reported from leaf and root transcriptomes by Gupta and co workers [7] while in the present study, 182 pathways were identified. Significant differences in the number of transcript contigs annotated under different secondary metabolite pathways were documented. In the present study, a total of 1354 transcript contigs was categorized under secondary metabolite pathways while 1068 unigenes were annotated from the previous study. A comparison of the number of transcript contigs/unigenes represented under the different secondary metabolite pathways across both the studies is given in table S3. This difference in transcript representation may be attributed to the total number of annotated transcripts, which significantly differed in both the studies.

Changes in ploidy level are known to significantly shape the plant genomes [54] and impact genetic and epigenetic aspects including gene expression [55], [56]. In *Arabidopsis thaliana*, altered gene expression was reported between autotetraploid and diploid ecotypes [57]. Similarly, in buffalo grass (*Buchloe dactyloides*), a significant difference in the number of transcripts assembled from the transcriptome data was documented between the tetraploid and hexaploid cultivar [58] suggesting that ploidy levels can impact the number of annotated transcripts during *de novo* assembly. In *W. somnifera*, considerable morphogenetic diversity is documented in Indian populations and intraspecific diploid (2n = 24), tetraploid (2n = 48) and hexaploid (2n = 72) cytotypes are reported [59]. The autotetraploid cytotype of this species was reported to yield higher root alkaloid in comparison to their diploid counterparts [60]. Hence, the difference in the number of annotated transcript contigs/unigenes between the two studies in *Withania* can also be attributed to a probable difference in ploidy level of the genotypes sequenced in the two studies. The ploidy level of the cytotype used in the present study was not determined while the same was not reported in the earlier study [7].

N50 is a statistical approach to assess the quality of the sequence assembly and higher N50 value indicate better assembly. The N50 of the present assembly was 1,978 bp suggesting optimum assembly and was higher than several plant transcriptome assemblies published earlier including *Coctus pictus* [61], *Cicer arietinum* [62], *Daucus carota* [63], *Cajanus cajan* [64] and *Euphorbia fischeriana* [65].

Discovery of EST-SSRs from transcriptome sequencing has significantly facilitated a cost effective method for identification of genic SSRs [66]. These SSRs can be used in genetic diversity analysis and for linkage/QTL/association mapping studies tagging traits of interest [67]. The higher rates of cross species transferability of EST-SSRs [68], [69] due to synteny across species has been demonstrated in rice [70], bread wheat [71], *Capsicum* [68], [72], sugarcane [73] and cotton [74]. In the present study, a total of 4250 SSRs were identified in comparison to the previous study [7] were a total of 2553 SSRs were reported in leaf transcriptome. Both studies registered higher number of tri- nucleotide repeats and minimum representation of penta- nucleotide SSRs. The abundance of tri-nucleotide repeats have been earlier reported from *Catharanthus roseus* [75], *Ipomoea batatas* [76] and *Leymus chinensis* [77].

In *Withania somnifera*, marker studies for diversity analysis are limited to RAPD, AFLP and ISSR [78], [79], [80]. Hence, the identification of EST-SSRs from the present study and the earlier report [7] will facilitate future studies on molecular markers in this species.

### Elicitor induced production of withanolides under culture conditions

The production of secondary metabolites under *in vitro* condition are reported to be enhanced by exogenous application of elicitors (biotic and abiotic) in culture media [81] and methyl jasmonate and salicylic acid are widely reported to induce production of secondary metabolites under culture conditions [82], [83]. In *W. somnifera*, the exogenous application of salacin was reported to induce the production of withaferin A in suspension cultures [84] while chitosan, methyl jasmonate and SA induced the production of withanolides in adventitious root and hairy root culture [83], [85], [86], [87]. Similarly, the present study recorded increase in production of three major metabolites of *W. somnifera* including withanoside V, withaferin A and withanolide A in leaf tissues, subsequent to exogenous application of SA.

### Selection of reference gene for qRT-PCR

Reliable quantification of gene expression levels by qRT-PCR requires the standardization and fine-tuning of several parameters, such as amount of initial sample, RNA recovery and integrity, enzymatic efficiencies of cDNA synthesis, PCR amplification and overall transcriptional activity of the tissues or cells analyzed [88]. Among various methods, internal control genes (reference genes) are most commonly used to normalize qRT-PCR data and reduce possible errors generated during quantification of gene expression [88], [89]. Nevertheless, this method relies on the choice of appropriate house-keeping genes, which ideally has stable expression under different experimental conditions and in different tissue types. In *W. somnifera*, there are no reports on selection of endogenous reference gene for normalization of qRT-PCR data under any experimental conditions or tissue types. In the earlier reports, actin was used as the reference gene for data normalization [90], [91]. However, in the present study actin was not identified as a stable gene for data normalization while *WsTUB* was documented to be the most suitable reference gene for quantitative gene expression studies. In members solanaceae family like potato, tobacco, tomato and *Capsicum annuum*, several house-keeping genes were screened to identify the most stable reference gene for a given experimental condition. In potato, *ef1α* and ribosomal protein (*L2*) was reported as the most stable gene during biotic and abiotic stress treatments and actin and tubulin where found to be least stable [92]. Similarly, in tobacco *ef1α* and *L25* was reported as most stable for qRT-PCR studies for developmentally distinct tissues and abiotic stresses [93]. However, in *Capsicum annuum*, beta tubulin and ubiquitin-conjugating protein showed high stability in sample pools with abiotic stress and hormonal treatments [94]. In tomato, the most stable reference gene for analyzing the gene expression during the interaction with the endophyte *Fusarium oxysporum* was *TUB* and *PP2ACS* for roots and *EF1* and *PP2ACS* for cotyledons [95]. These studies highlight that use of universal reference gene for qRT-PCR may not be ideal for data normalization. Hence, screening of stable reference genes for a given tissue type and experimental condition is a pre-requisite for data validation. The present study in *Withania* is the first report on identification of stable reference gene, which can support future gene expression studies in this important medicinal plant.

### Expression of SAIGs during SA signaling

Salicylic acid is a phenolic compound which plays a central role in plant defense signaling network [96]. It is important for basal defense, protein- mediated defense and systemic acquired resistance [97], [98], [99], [100]. SA-mediated immune response is integral part of both PAMP-triggered and effector-triggered immunity [100] and also a prerequisite for activation of SAR [101]. Earlier studies have indicated that pathogen infection leads to SA accumulation both in locally infected tissues and distal uninfected tissues that develop SAR [102], [103] and concurrently results in up-regulation of PR genes [104]. Studies have revealed that SA also plays an important role in controlling the cellular redox balance at the onset of SAR [105], [106]. The SA associated gene expression has been grouped into three categories in *Arabidopsis*, type I including genes encoding enzymes that are directly involved in SA biosynthesis, type II including proteins that do not act directly on SA biosynthesis but mutations in these genes lead to compromised SA accumulation and disease susceptibility and type III including genes which act downstream of SA accumulation like *NPR1*, a major signal transducer of SA and PR genes [107], [108]. Exogenous application of SA can mimic the endogenous increase that occurs during pathogen infection and elicit SAR.

The PR proteins/genes which are considered signatures of the SA signaling are PR1, PR2 and PR5 [109], [110]. SA signaling mutants and transgenics expressing bacterial salicylate hydroxylase with reduced SA accumulation have impaired ability of SAR and reduced expression of PR1, PR2 and PR5 [111], [112], [113]. In *Arabidopsis*, several mutants with impaired disease response have been developed to understand the signaling pathways operational during pathogenesis. Mutant phenotypes with increased SA levels including constitutive immunity (*cim*) [114], constitutive expression of PR proteins (*cpr*) [115], [116] and defense no cell death (*dnd1*) [117] recorded higher expression levels of PR genes. Similarly, mutants with impaired SA accumulation like *pad4* [118] and SA induction–deficient (*sid*) [119] documented low expression of PR1 and increased disease symptoms, reiterating the predominant role of SA in disease resistance and induction of PR genes/proteins. In the present study the temporal expression of 17 genes representing 12 pathogenesis-related (PR) families were analyzed during SA signaling.

Plant chitinases classified under PR protein families PR3, PR4, PR8 and PR11 [120], [121] include one of the most characterized families of PR proteins which catalyze the hydrolysis of chitin present in fungal cell wall and exoskeleton of insects. The induction of different classes of chitinases during exogenous application of SA was reported in *Pinus elliottii* [122]; cucumber [123]; cotton [124]; *Castanea sativa* [125]; tobacco [126]; sweet cherry [127], [128], grape berries [129], sorghum [130], *Casuarina equisetifolia* [131], *Malus hupehensis* [22] and tomato [132]. In *Vitis vinifera*, two classes of chitinases (Class I and Class III) were analyzed for their expression during SA mediated SAR and results revealed that the class III chitinase expressed in distal leaves, suggesting it as a reliable indicator of SAR [133]. Similarly, in the present study, a significant up-regulation of three chitinases including *WsCHTN2, WsCHTN3* and *WsCHTN4* and moderate induction of *WsCHTN5* was documented. However, the present study also documented the down-regulation of a class I chitinase (*WsCHTN1*) after 17 hours of SA treatment. In concurrence to the present result, study in *Vitis vinifera* revealed no significant change in expression of class I chitinase when challenged with SA [133].

Beta-1,3 glucanase classified under PR-2 play a direct role in fungal defense by hydrolyzing the fungal cell wall and an indirect role by generating oligosaccharide elicitors [134]. In the present study, this gene (*WsB13G*) was up-regulated by 287 fold after 36 hours of SA treatment. Similarly, earlier studies where exogenous application of SA induced expression of PR-2 are reported from tomato [132], *Eucalyptus grandis* [135], *Casuarina equisetifolia* [136], cotton [124], sweet cherry [127], [128], grape berries [129] and tobacco [126]. However, down-regulation of PR-2 is also reported in sorghum [130] and wheat [34] during SA signaling and pathogen infection.

PR-1 is induced by pathogens and salicylic acid and is commonly identified as a marker for SAR. Their antifungal activity suggests involvement in plant defense, but their mode of action or relationship to other proteins is unknown. In *W. somnifera*, *WsPR1* was up-regulated by 21-fold subsequent to 36 hours of SA treatment. Similarly, induction of PR-1 was reported during SA treatment from *Arabidopsis* [36], [137], tomato [138] and tobacco [126], [139]. In *Malus hupehensis* seedlings, application of SA enhanced the expression of *MhPR1, MhPR5* and *MhPR8* after 48 hours of post –induction [22].

Peroxidases (PR-9) are heme-containing oxido-reductases and its activities have been correlated with plant resistance. They are involved in the oxidation of phenolic residues to cell wall polymers in pathogen-infected tissues. In the present study, two peroxidases *viz*., lignin-forming and suberization-associated anionic peroxidase was induced by 377-fold and 6532-fold on SA application. Similarly, up-regulation of peroxidases is reported from sorghum [130], sunflower [141], *Vigna unguiculata* [140] and *Polygonum minus* [142]. In woody perennials like *Pyrus bretschneideri cv. aYali*, exogenous application of SA induced the accumulation of several PR proteins including chitinase, glucanase and peroxidase. Further, the SA treated leaves showed reduced symptoms of ring rot disease caused by *Physalospora piricola* [143]. The increased peroxidase activity could be due to the increased lignin biosynthesis, which forms the basal defense response in plants [144].

PR-10 proteins including pollen allergens are present as multigene family in seed plants and are developmentally and environmentally regulated. PR-10 proteins are reported from numerous dicots, including parsley, pea, potato, bean, soybean, celery and alfalfa and monocots like asparagus, rice, lily and sorghum [145]. PR-10 from *W. somnifera* (*WsPR10*) was down-regulated by 8-fold and 14-fold after 17 and 36 hours of SA application respectively. Similarly, in western white pine, the wound inducible *PmPR10* transcript was partially suppressed by SA [145]. The expression of root specific PR-10 induced by drought and salt (*RSOsPR10*) was strongly inhibited by SA treatment in rice roots [146]. However, up-regulation of PR-10 has been reported from *Arabidopsis* [137], sorghum [130], soybean [147], asparagus [148], *Medicago sativa* [149], bean [150], rice [151], [152], [153] and *Lithospermum erythrorhizon* [154] during SA signaling and pathogenesis. This revealed that different signal transduction pathways might be involved in activation of different classes of PR10 to different environmental stresses [145].

The other PR genes which were up-regulated after 36 hours of SA treatment in *Withania* included thaumatin-like *WsTHAU* (PR5), *WsCYST* and *WsSPI*, (PR6), *WsDEFN* (PR12), lipid transfer proteins (PR14) and germin-like *WsGER1* (PR16). Concurrently, the up-regulation of thaumatin-like PR-5 during SA treatment was reported from *Malus hupehensis* [22], sorghum [130], wheat [155], *Arachis diogoi* [156] and *Eucalyptus grandis* [135].

The synthetic analogs of SA including Benzothidiazole, benzo (1,2,3) thiadiazole-7-carbothioic acid S-methyl ester (BTH), 2,6-dichloroisonicotinic acid (INA) and 2,6-dichloroisonicotinic acid (DCINA) are chemical inducers of SAR and are commercially used to induce resistance to pathogenic infection in crops [157]. These functional analogs are also known to induce PR proteins/genes [26], [158], [159]. In sugar beet, BTH induced accumulation of chitinase and glucanase [160] while in soybean, the chemical up-regulated expression of PR-1, PR-3a, PR-3b, PR-9, and PR- 10 [157]. In banana, exogenous application of BTH caused prolonged expression of chitinase and reduced the symptoms of anthracnose disease [161]. Similarly, exogenous application of aspirin and BTH induced the expression of PR10 in *Lithospermum erythrorhizon* [154]. In sunflower, acetylsalicylic acid (asprin) induced the expression of four PR proteins including PR1, PR2, PR3 and PR5 which comprised 80% of the intercellular fluid proteins of induced leaf discs [20]. In barley seedlings, exogenous application of DCINA induced disease resistance against *Erysiphe graminis f. sp. hordei* and the acquired resistance was associated with increased accumulation of PR transcripts including PR1, chitinase and peroxidase [162]. Similarly, up-regulation of *CaPR1, CaPR4, CaPR9* and *CaCHI2* was reported in pepper during elicitation by BTH [23], while in maize, PR1 and PR5 were induced by BTH and INA. [163].

Recently, with the introduction of cost-effective NGS platforms for transcriptome sequencing, studies on understanding the global gene expression patterns during pathogenesis are being undertaken in several plant species. Host transcriptome analysis during interaction with pathogens are reported in banana - *Fusarium oxysporum* [32], *Musa acuminate* - *Mycosphaerella musicola* [33], wheat - *Fusarium graminearum* [34], potato- *Phytophthora infestans* [35], peach - *Xanthomonas arboricola* pv. *Pruni* [37] and *Lactuca sativa* - *Botrytis cinerea* [38]. These studies have also highlighted the up-regulation of PR gene families during pathogenesis.

The results on expression patterns of different PR genes during SA treatment documented in the present study and earlier studies from other plant species indicate that most of the pathways mediated by SA are analogous, but gene expression patterns can be species/genotype specific.

The present study is an attempt to characterize the SA mediated transcriptome in *W. somnifera*, a non-model medicinal species. The data generated in this study can support future studies in understanding the transcriptional regulation and networking of different pathways during pathogen defense response in *Withania* and other allied species from the Solanaceae family.

## Supporting Information

Figure S1 Effect on different concentration of salicylic acid on leaf discs of *W. somnifera*.
(TIF)

Figure S2 E-value distribution of transcript contigs from RNA-Seq data of *W. somnifera*.
(TIF)

Figure S3 Sequence similarity distribution of transcript contigs from RNA-Seq data of *W. somnifera*.
(TIF)

Figure S4 GO mapping of transcript contigs from RNA-Seq data of *W. somnifera* to different databases.
(TIF)

Figure S5 Evidence code distribution of transcript contigs (A) and annotated transcript contigs (B) from RNA-Seq data of *W. somnifera*.
(TIF)

Figure S6 Annotation score distribution of transcript contigs from RNA-Seq data of *W. somnifera*.
(TIF)

Figure S7 GO-level-wise sequence distribution of transcript contigs for (a) Biological processes (b) Molecular functions (c) Cellular components. The initial levels denote the general function of transcript contigs and with progression of levels the function of the transcript contigs becomes more specific. Each transcript contigs can be multi-functional and hence, can lie in more than one ontology domain.
(TIF)

Figure S8 Melt curve analysis of reference genes used for normalization of qRT-PCR data.
(TIF)

Figure S9 Melt curve analysis of PR genes used for expression profiling.
(TIF)

Table S1 Annotation of *W. somnifera* leaf transcriptome using Nr database.
(XLSX)

Table S2 Classification of transcript contigs to biological pathways in *W. somnifera* using KEGG database.
(XLS)

Table S3 Comparison of number of transcript contigs represented under different secondary metabolite pathways in two independent studies conducted on *W. somnifera*.
(DOC)
