# Supplementary figures and images for: Characterization of Withania somnifera Leaf Transcriptome and Expression Analysis of Pathogenesis – Related Genes during Salicylic Acid Signaling
Source: PLoS One. 2014 Apr 16;9(4):e94803. doi: 10.1371/journal.pone.0094803 (PMC3989240; doi:10.1371/journal.pone.0094803)

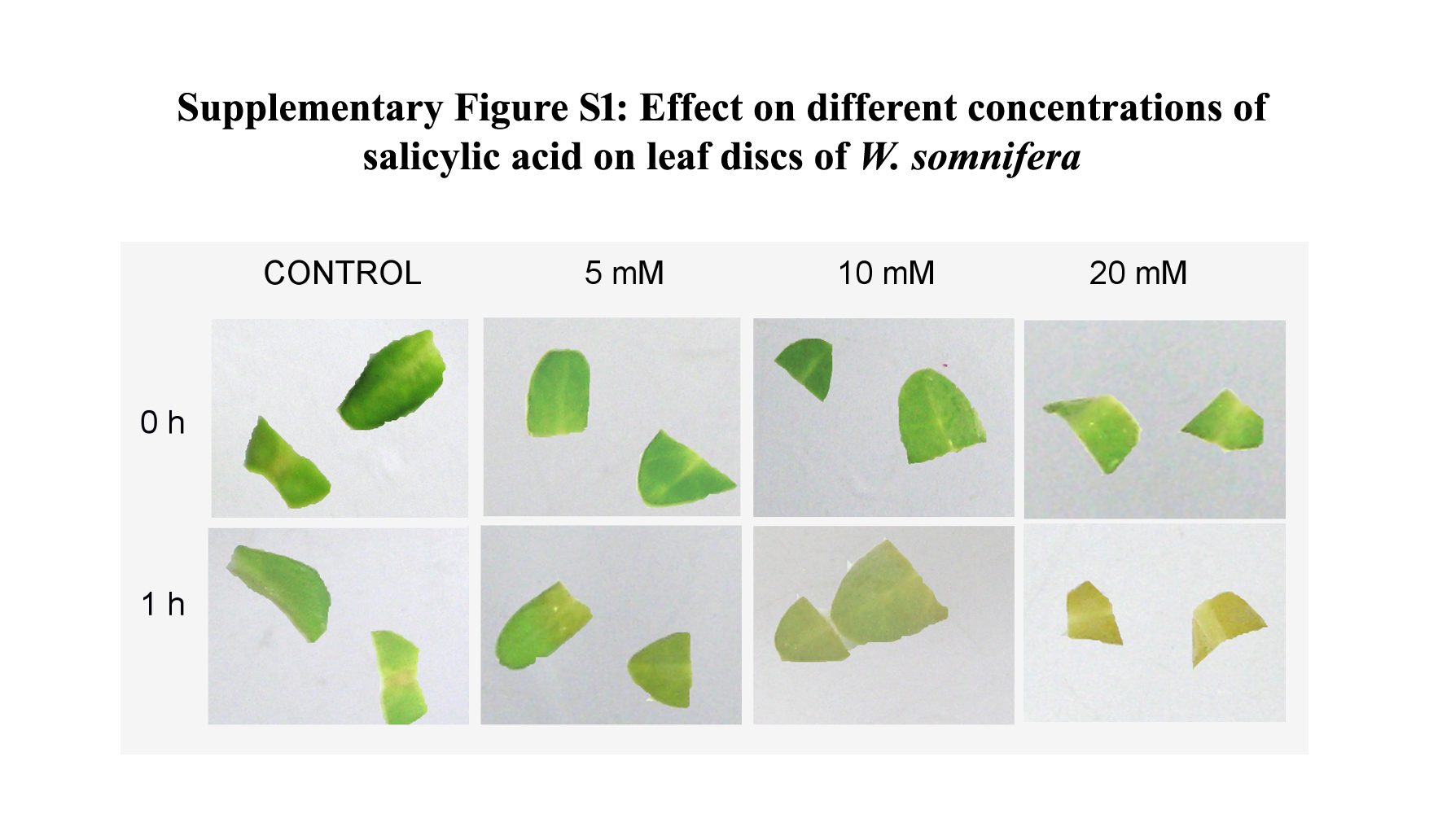

Supplement: Figure S1 — Effect on different concentration of salicylic acid on leaf discs of W. somnifera. (TIF) [file pone.0094803.s001.tif]

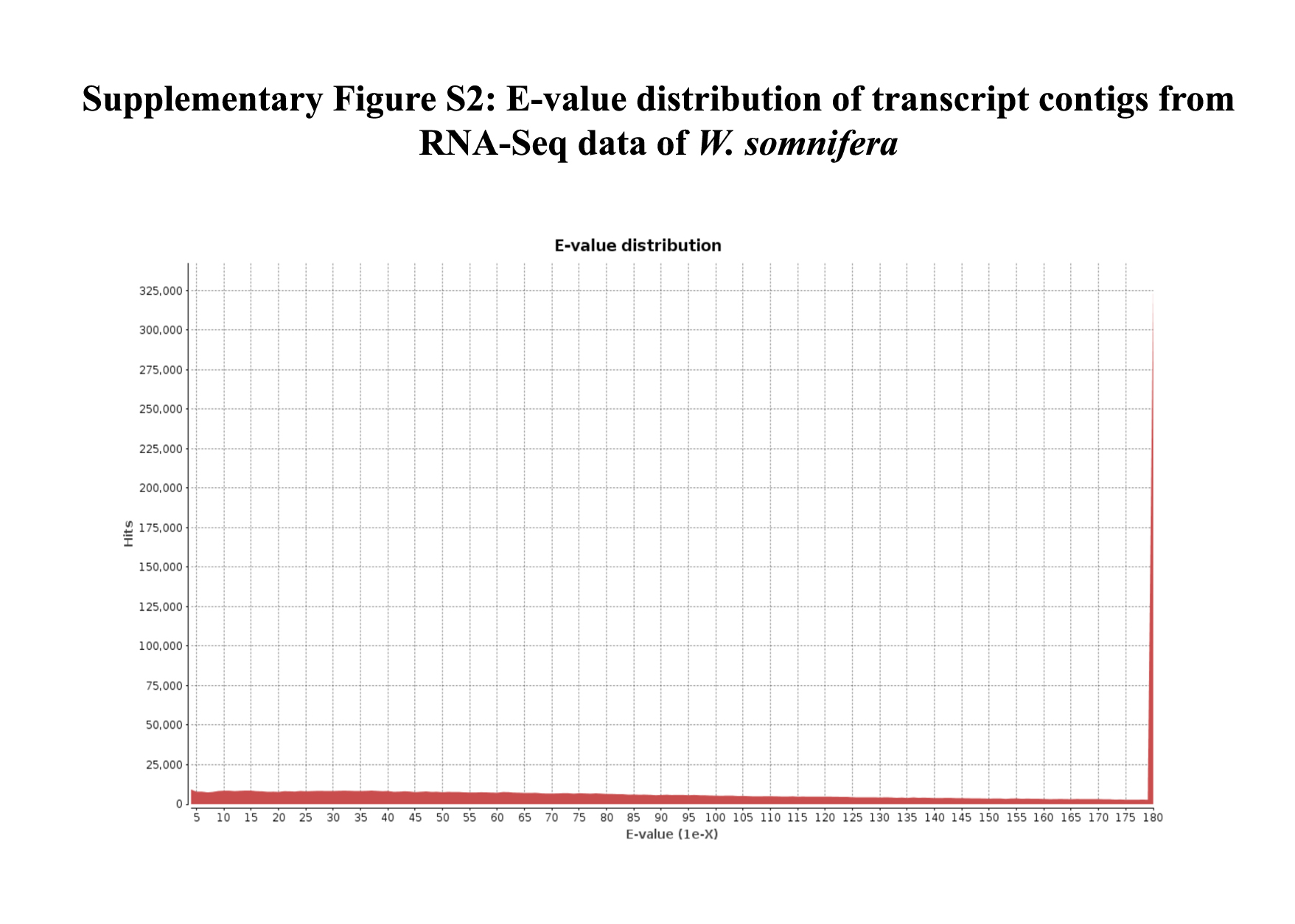

Supplement: Figure S2 — E-value distribution of transcript contigs from RNA-Seq data of W. somnifera. (TIF) [file pone.0094803.s002.tif]

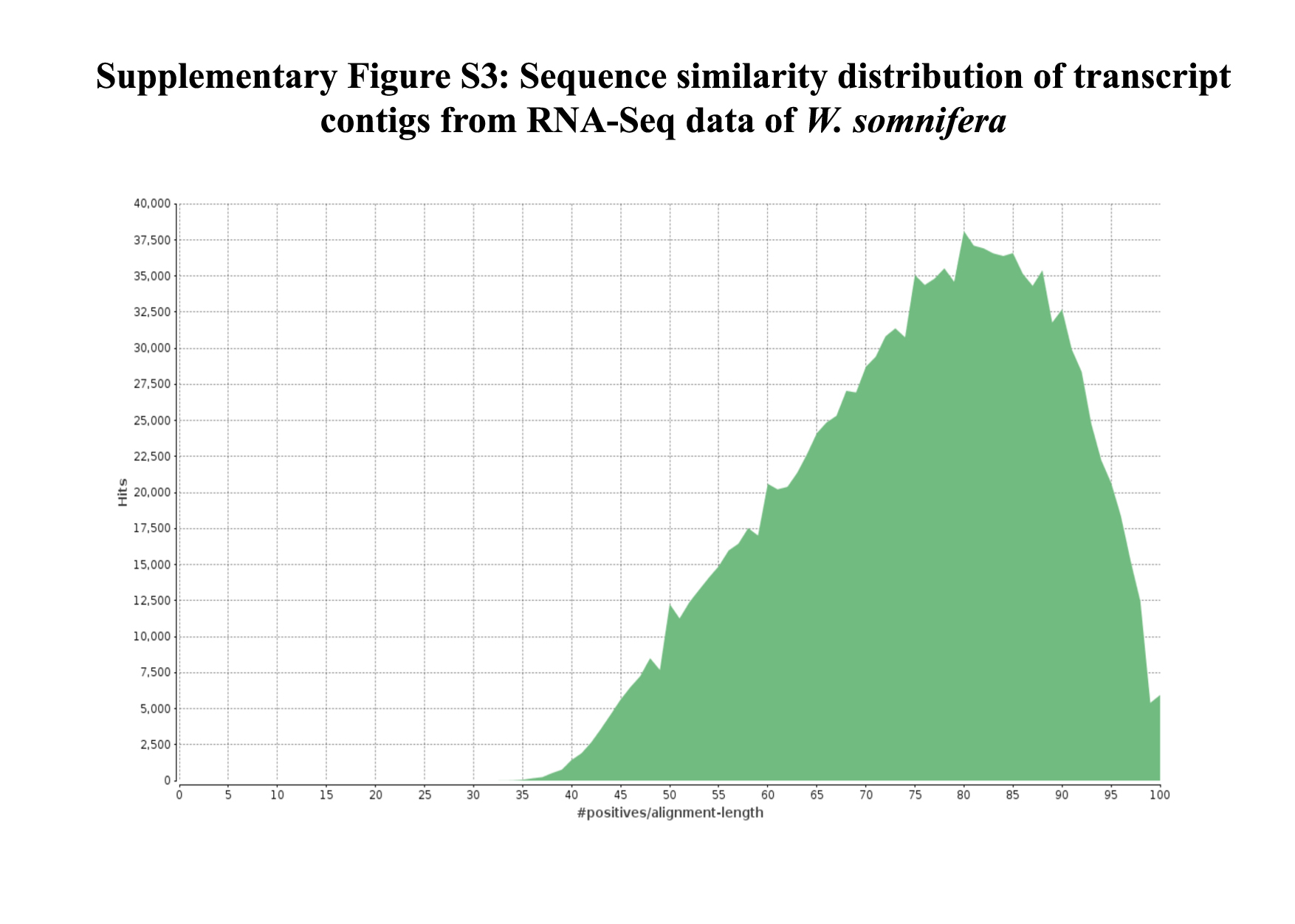

Supplement: Figure S3 — Sequence similarity distribution of transcript contigs from RNA-Seq data of W. somnifera. (TIF) [file pone.0094803.s003.tif]

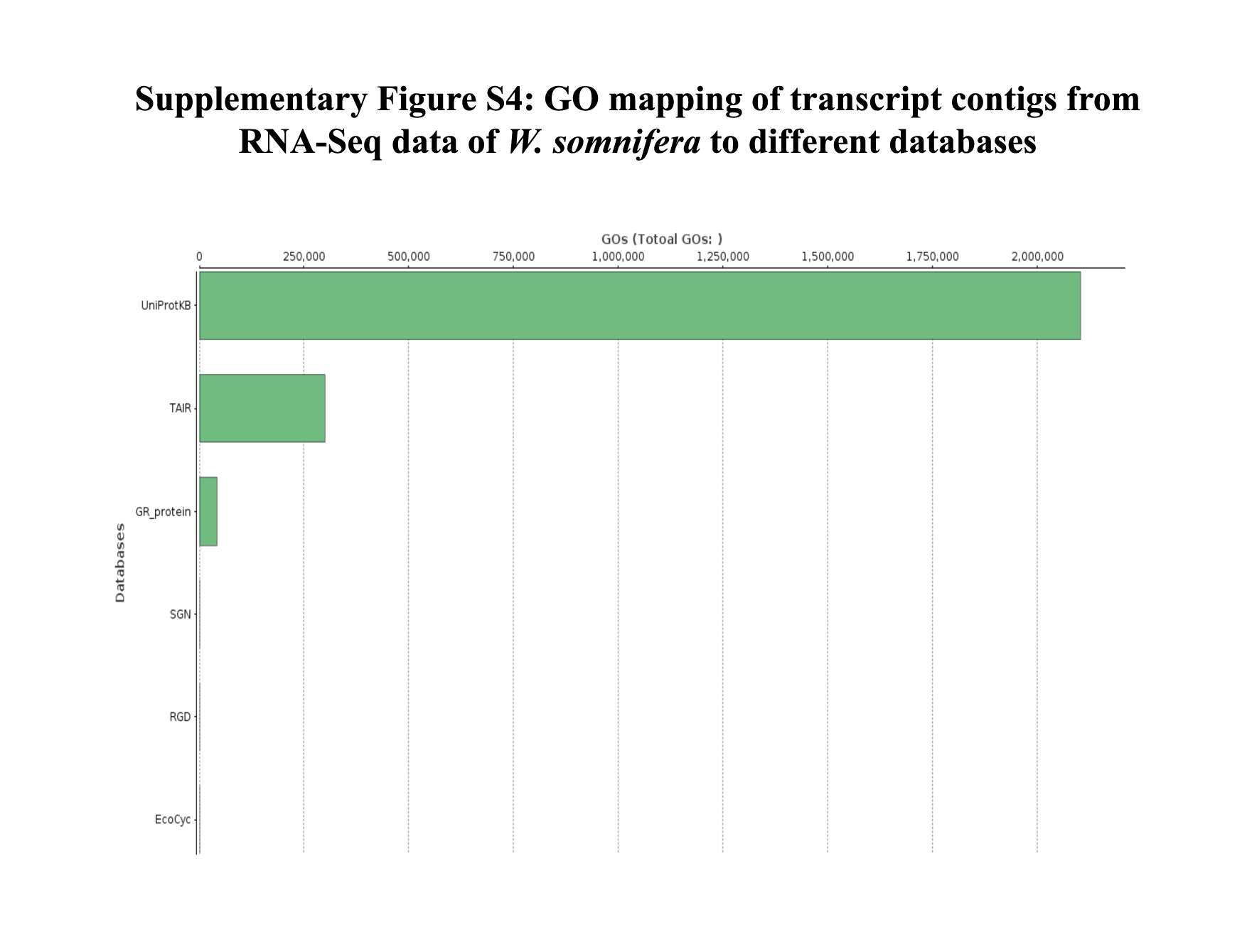

Supplement: Figure S4 — GO mapping of transcript contigs from RNA-Seq data of W. somnifera to different databases. (TIF) [file pone.0094803.s004.tif]

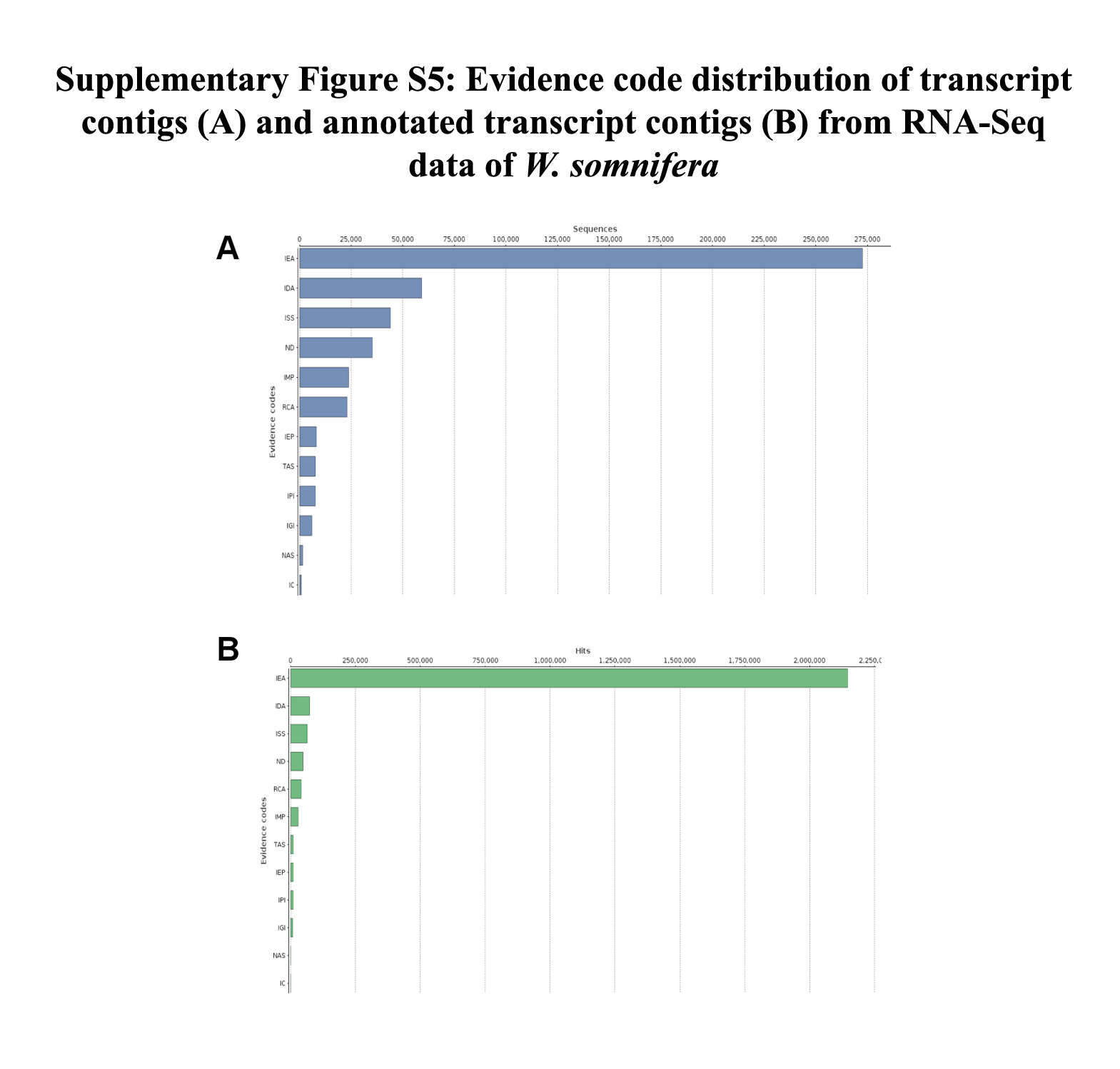

Supplement: Figure S5 — Evidence code distribution of transcript contigs (A) and annotated transcript contigs (B) from RNA-Seq data of W. somnifera. (TIF) [file pone.0094803.s005.tif]

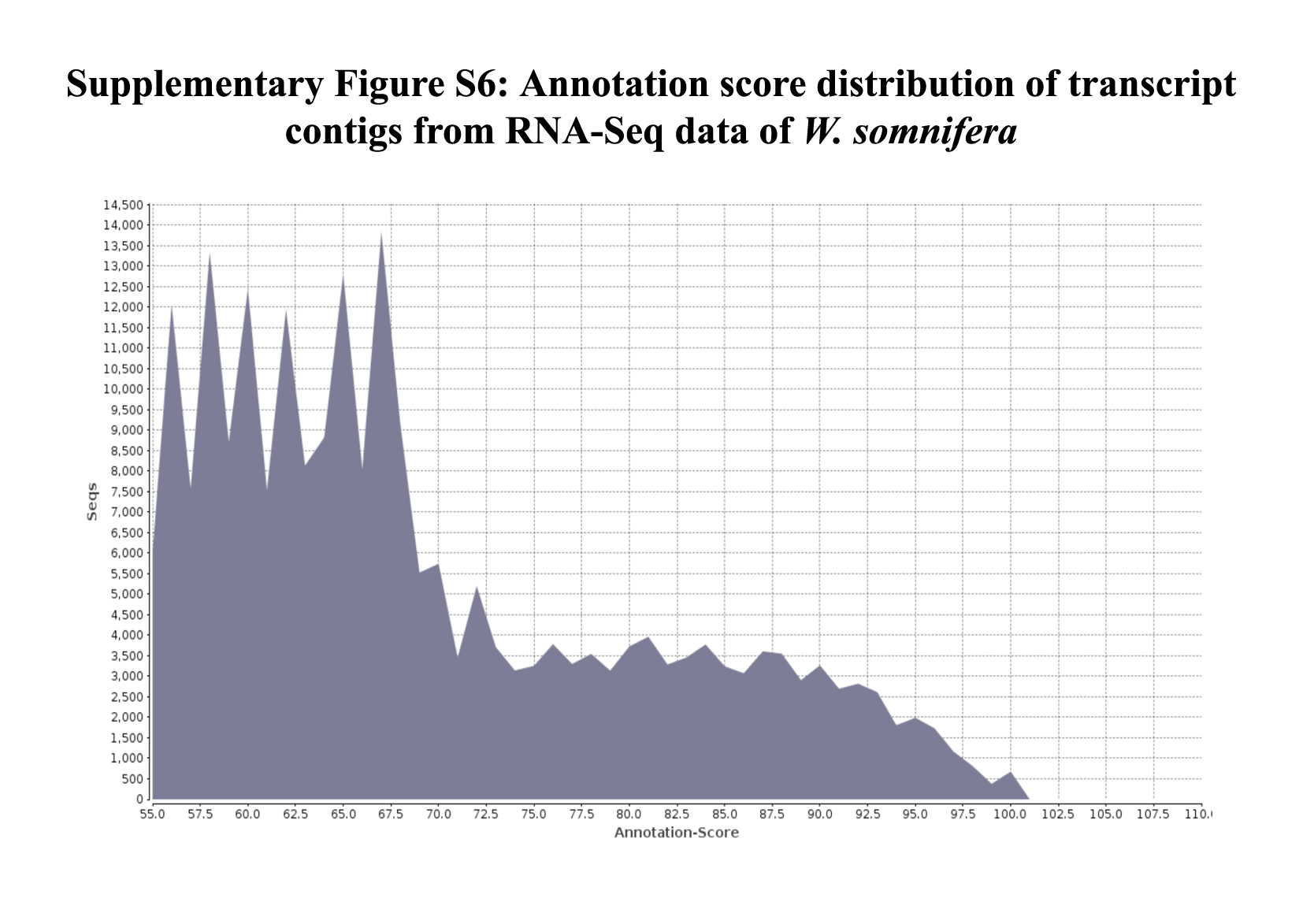

Supplement: Figure S6 — Annotation score distribution of transcript contigs from RNA-Seq data of W. somnifera. (TIF) [file pone.0094803.s006.tif]

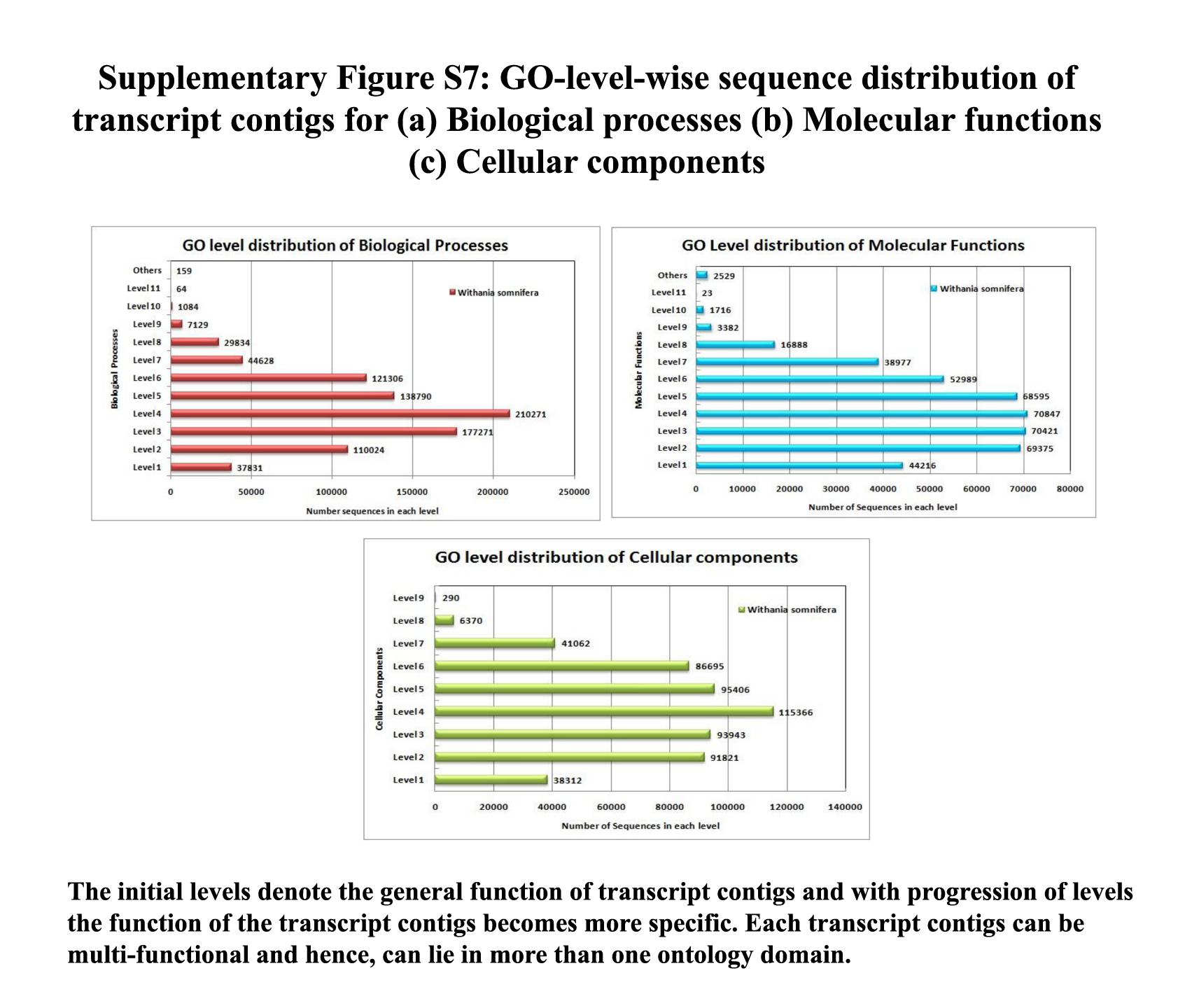

Supplement: Figure S7 — GO-level-wise sequence distribution of transcript contigs for (a) Biological processes (b) Molecular functions (c) Cellular components. The initial levels denote the general function of transcript contigs and with progression of levels the function of the transcript contigs becomes more specific. Each transcript contigs can be multi-functional and hence, can lie in more than one ontology domain. (TIF) [file pone.0094803.s007.tif]

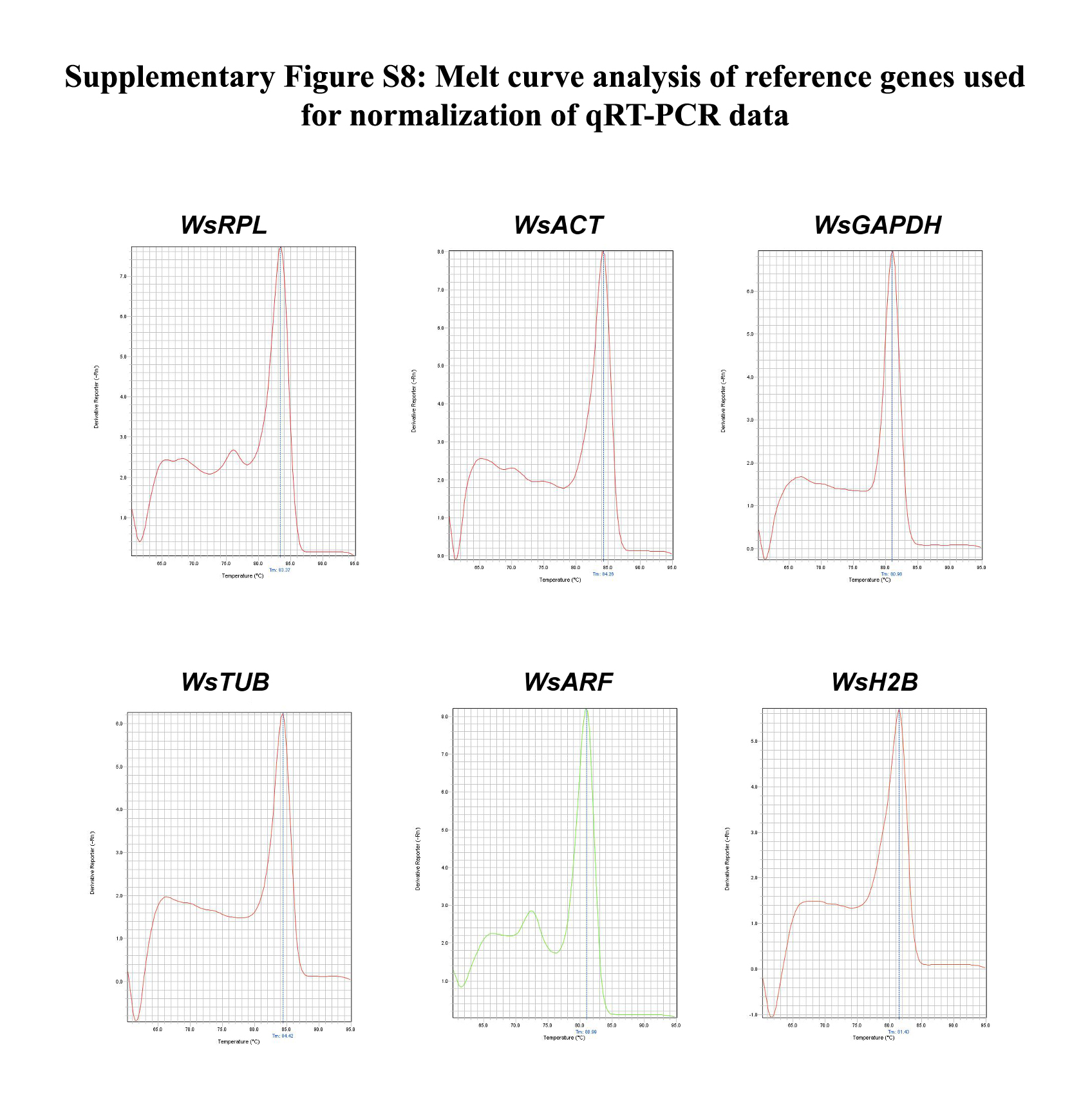

Supplement: Figure S8 — Melt curve analysis of reference genes used for normalization of qRT-PCR data. (TIF) [file pone.0094803.s008.tif]

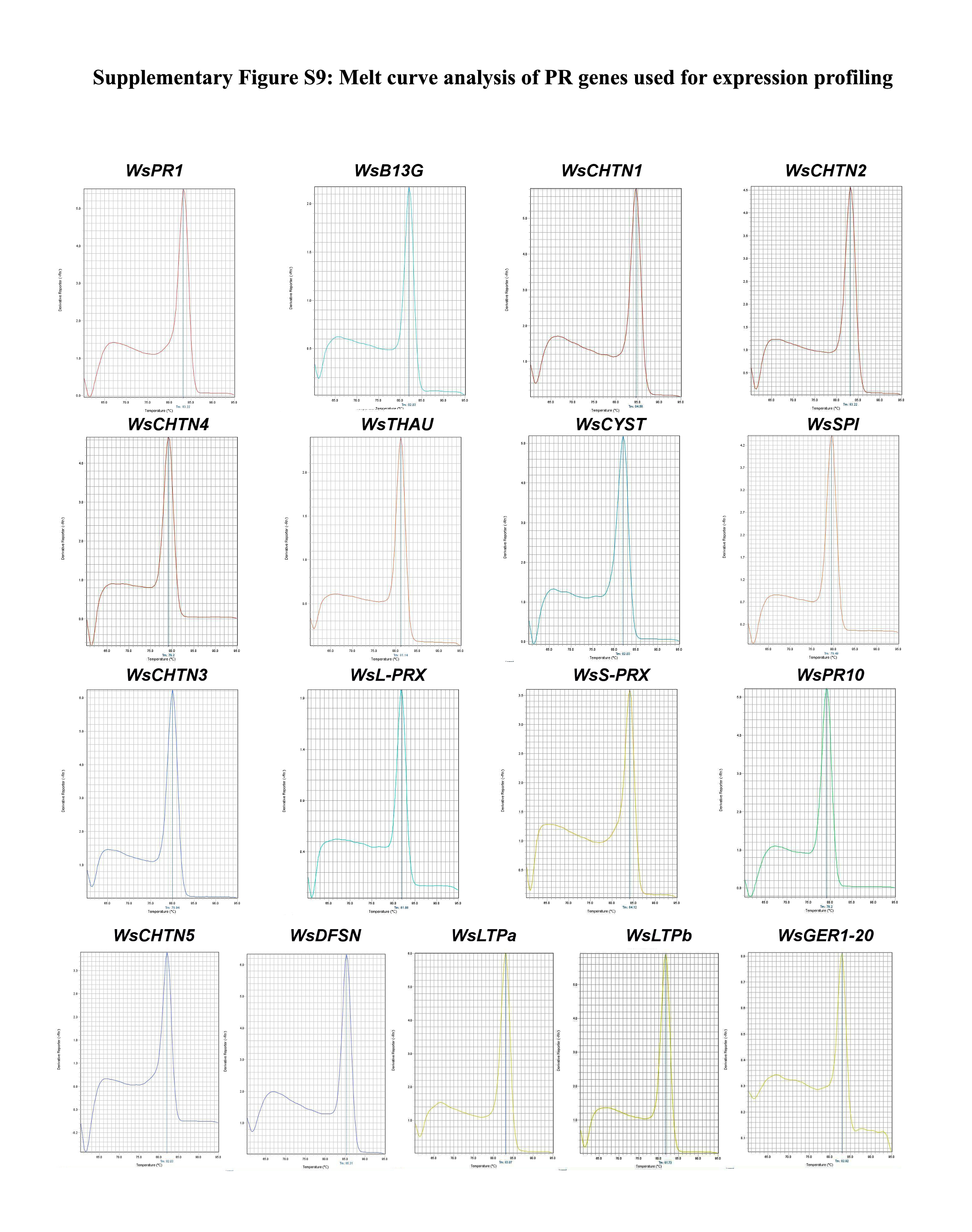

Supplement: Figure S9 — Melt curve analysis of PR genes used for expression profiling. (TIF) [file pone.0094803.s009.tif]
