# Supplementary material for: Characterization of Withania somnifera Leaf Transcriptome and Expression Analysis of Pathogenesis – Related Genes during Salicylic Acid Signaling
Source: PLoS One. 2014 Apr 16;9(4):e94803. doi: 10.1371/journal.pone.0094803 (PMC3989240; doi:10.1371/journal.pone.0094803)
Supplement: Table S3 — Comparison of number of transcript contigs represented under different secondary metabolite pathways in two independent studies conducted on W. somnifera. (DOC) [file pone.0094803.s012.doc]

***Supplementary Table S3: Comparison of number of transcript contigs represented under different secondary metabolite pathways in two independent studies conducted on*** W. somnifera

| **Secondary metabolite pathways** | **Present study** | **Gupta et al. (2013b)** |
| --- | --- | --- |
| Terpenoid backbone biosynthesis [PATH: 00900] | 204 | 124 |
| Monoterpenoid biosynthesis [PATH: 00902] | 8 | 9 |
| Sesquiterpenoid and triterpenoid biosynthesis [PATH: 00909] | 32 | 50 |
| Diterpenoid biosynthesis [PATH: 00904] | 43 | 25 |
| Carotenoid biosynthesis [PATH: 00906] | 112 | 80 |
| Brassinosteroid biosynthesis [PATH: 00905] | 15 | 15 |
| Zeatin biosynthesis [PATH: 00908] | 49 | 26 |
| Limonine and pinene degradation [PATH: 00903] | 70 | 92 |
| Phenylpropanoid metabolism [PATH: 00940] | 287 | 192 |
| Stibenoid diarylhepatanoid and gingerol biosynthesis [PATH: 00945] | 94 | 82 |
| Flavonoid biosynthesis [PATH: 00941] | 66 | 48 |
| Flavone and flavonol biosynthesis [PATH: 00944] | 28 | 43 |
| Anthocyanin biosynthesis [PATH: 00942] | 7 | 5 |
| Indole alkaloid biosynthesis [PATH: 00901] | 3 | 8 |
| Isoquinoline alkaloid biosynthesis [PATH: 00950] | 22 | 42 |
| Tropane, piperidine and pyridine alkaloid biosynthesis [PATH: 00960] | 37 | 39 |
| Caffeine metabolism [PATH: 00232] | 8 | 15 |
| Cutin, suberin and wax biosynthesis [PATH: 00073] | 23 | 10 |
| Steroid biosynthesis [PATH: 000100] | 73 | 54 |
| Nicotinate and nicotinamide metabolism [PATH: 00760] | 84 | 36 |
| Ubiquinone and other terpenoid-quinone biosynthesis [PATH: 00130] | 89 | 73 |
| TOTAL | 1354 | 1068 |
